# Outcomes of Laparoscopic Radical Hysterectomy in Ia1-Ib1 Cervical Cancer Patients: A Multi-Center Study with 10 Years’ Experiences in the Real World

**DOI:** 10.1245/s10434-024-16637-3

**Published:** 2024-12-29

**Authors:** Chenge Zhang, Wenfang Tian, Xiaofang Zhou, Lesai Li, Shanmei Tan, Lijuan Sun, Jie Tang

**Affiliations:** 1 Department of Gynecologic Oncology, The Affiliated Cancer Hospital of Xiangya School of Medicine Central, South University/Hunan Cancer Hospital, Changsha, People’s Republic of China; 2 Department of Gynecology and Obstetrics, Huaihua No. 1 People’s Hospital, The Affiliated Huaihua Hospital of University of South China, Huaihua, People’s Republic of China; 3 Department of Gynecology and Obstetrics, Central Hospital of Shaoyang, Shaoyang, People’s Republic of China; 4 Department of Gynecology and Obstetrics, Hengyang Medical School, Graduate Collaborative Training base of Hunan Cancer Hospital, University of South China, Hengyang, People’s Republic of China

**Keywords:** Low-risk cervical cancer, Laparoscopy, Overall survival, Progression-free survival, Radical hysterectomy

## Abstract

**Background:**

The aim of this retrospective study was to evaluate the outcomes of laparoscopic radical hysterectomy (LRH) for International Federation of Gynecology and Obstetrics (FIGO) 2018 stage IA1 IB1 patients with low-risk cervical cancer (CC), which was defined as tumor ≤ 2cm, less than 1/2 stromal invasion and no lymph node involvement.

**Patients and Methods:**

We performed a retrospective analysis of patients with CC who underwent radical hysterectomy across three hospitals between 2010 and 2020. The patients were stratified into low-risk and high-risk groups based on risk factors (tumor size, lymph nodes and stromal invasion depth). Within each group, the survival outcomes of open abdominal radical hysterectomy (OARH) and LRH were compared using the Kaplan–Meier analysis.

**Results:**

In the low-risk group (LRH: *N* = 320; OARH: *N* = 525), LRH demonstrated equivalence to OARH regarding 5-year overall survival (OS; 98.6% versus 99.3%, *P* = 0.571) and 5-year progression-free survival (PFS; 97.6% versus 98.4%, *P* = 0.418). Subsequently, a stratified analysis based on lymphovascular space invasion (LVSI) status revealed no significant differences in 5-year OS and PFS between LRH and OARH in this group. Conversely, in the high-risk group (LRH: *N* = 355; OARH: *N* = 926), LRH exhibited significantly lower 5-year OS and PFS than OARH (91.3% versus 94.8%, *P* = 0.049; 84.0% versus 88.8%, *P* = 0.029).

**Conclusion:**

Among FIGO 2018 stage IA1–IB1 patients with low-risk CC, LRH demonstrates survival outcomes comparable to OARH. For patients with early-stage and low-risk CC, the appropriate surgical approach (LRH) can be chosen based on preoperative enhanced magnetic resonance imaging (MRI) and diffusion-weighted imaging (DWI) MRI, which is clinically feasible.

Cervical cancer (CC) ranks as the fourth most common malignancy and is a leading cause of mortality among women globally.^1^ Radical hysterectomy accompanied by pelvic lymphadenectomy stands as the standard treatment for early-stage CC; however, the surgical approach (open abdominal or minimally invasive approach) has recently come under question.

Since the advent of laparoscopic radical hysterectomy (LRH) as a treatment for cervical cancer in 1992,^2^ a multitude of retrospective studies have indicated that this procedure offers a diminished risk of infection, expedited recovery, and comparable oncological outcomes with open abdominal radical hysterectomy (OARH).^3–11^ Consequently, the last two decades have seen a significant increase in the preference for LRH in managing CC.

However, in 2018, *The New England Journal of Medicine* published a cohort study conducted by Melamed A, which revealed that minimally invasive radical hysterectomy (MIS) corresponded with shorter overall survival compared with open surgery in women with early-stage CC.^12^ Meanwhile, the laparoscopic approach to cervical cancer (LACC) trial—a phase III, multicenter, randomized study—reported that MIS was tied to a fourfold increase in the risk of recurrence and a sixfold escalation in the risk of death relative to OARH in women with early-stage CC.^13^ These two seminal studies prompted the National Comprehensive Cancer Network (NCCN) to revise their guidelines and recommending open radical hysterectomy as the standard surgical approach for early-stage CC.^14^

By carefully studying the results of LACC trial, we were surprised to find that there were less than 30% of patients with early-stage, low-risk CC, which was defined as tumor ≤ 2cm, less than 1/2 stromal invasion, and no lymph node involvement. So, in this study, we carried out a retrospective analysis of real-world cases in three hospitals over a decade to assess the oncological outcomes of LRH in patients with early-stage, low-risk CC.

## Patients and Methods

### Inclusion and Exclusion Criteria

**Fig. 1:**
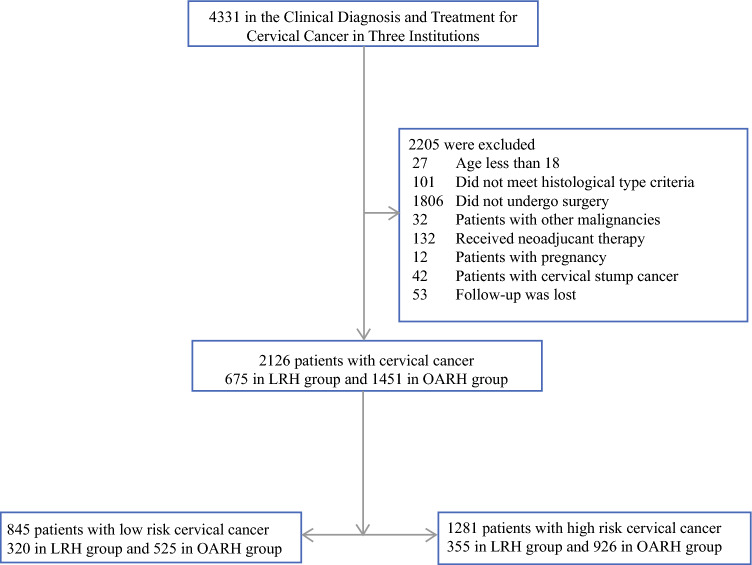
Flow diagram of recruitment and exclusions. *OARH* open abdominal radical hysterectomy, *LRH* laparoscopic radical hysterectomy

This retrospective, multicenter study evaluates patients with cervical cancer who underwent clinical diagnosis and treatment at three institutions from January 2010 to September 2020. The inclusion criteria were as follows: (1) patient age of 18 years or older; (2) histological diagnosis of squamous cell carcinoma, adenocarcinoma, or adenosquamous carcinoma; (3) the surgical method employed was either abdominal or laparoscopic; (4) performance of an upfront Querleu–Morrow (Q–M) type B or type C radical hysterectomy with or without pelvic lymphadenectomy, with or without para-abdominal aortic lymphadenectomy; and (5) absence of neoadjuvant radiation or chemotherapy prior to surgery. The exclusion criteria were as follows: (1) patients did not undergo surgery; (2) patients who received preoperative adjuvant treatment; (3) pregnant patients; (4) patients with cervical stump cancer; (5) patients with concurrent malignancies; and (6) patients with histological types other than those specified. Our study was approved by the institutional review boards of Hunan Cancer Hospital. The criteria for participant inclusion are delineated in Fig. 1.

### Observation Index Follow-Up

Clinical data were collected from patient files and the medical record management system in the hospitals by trained gynecological oncology staff using standardized data collection and quality control procedures. The following data were collected: demographic information, operation year, histological type, clinical stage (International Federation of Gynecology and Obstetrics; FIGO), tumor size (image examination, gynaecologic examination, or pathology report), pathological results, stromal invasio, parametrial invasion, lymphovascular space invasion (LVSI), surgical margin, and adjuvant treatment condition. Preoperative evaluation of lymph node metastasis uses enhanced magnetic resonance imaging (MRI) and and diffusion-weighted imaging (DWI) MRI, with positron emission tomography (PET) computed tomography (CT) added if necessary. And all the patients were re-staged according to the FIGO 2018 criterion. The follow-up procedures were conducted by professional gynecologists through telephone or the return visit system, and the patient’s personal data were kept confidential. Follow-up information, included the survival status, time of death, recurrence time, recurrence site, and treatment after recurrence.

### Definition

The low-risk group was characterized by patients diagnosed with cervical cancer exhibiting tumor lesions ≤ 2 cm, negative lymph nodes, and less than 1/2 stromal invasion.^15–17^ Conversely, the high-risk group comprised patients with one or more moderate-to-high risk factors: positive lymph node metastasis, deep cervical stromal invasion (>50%), tumor size greater than 2 cm, or FIGO stage ≥ IB2.

The primary aim of the study was to compare the 5-year overall survival (OS) and 5-year progression-free survival (PFS) between OARH and LRH in the low-risk group. Secondary objectives included comparing the 5-year OS and PFS between OARH and LRH in the high-risk group, as well as determining complications across the two group. The PFS was delineated as the duration from the surgical operation to the date of cervical cancer recurrence. Similarly, OS was defined as the interval from the surgical procedure to the date of death from any cause. Patients with no evidence of recurrence or death were defined by the date of the last follow-up date or the last outpatient visit.

### Statistical Analysis

Measurements are presented as mean ± standard deviation (M ± SD) and are categorized as percentages (%). The Mann–Whitney *U* test was employed to compare continuous variables, while categorical variables were assessed using the chi-squared (*χ*^2^) test or Fisher’s exact test. Survival outcomes were depicted using Kaplan–Meier curves. Additionally, multivariate Cox proportional hazards regression analyses were conducted to estimate the hazard ratios (HRs) and 95% confidence intervals (CIs) for the survival outcomes of patients with cervical cancer. The statistical analyses were executed utilizing IBM SPSS Statistics version 25.0 (IBM Corp, Armonk, NY, USA). A *P* value of less than 0.05 was deemed indicative of statistical significance.

## Results

### Characteristics of the Patients

Between 2010 and 2020, altogether 4331 patients were diagnosed and treated for cervical cancer in three institutions. A total of 2126 patients met the above inclusion criteria and were included in the final analysis with 675 undergoing laparoscopic radical hysterectomy and 1451 undergoing open abdominal radical hysterectomy. All participants had a median follow-up of 49 months (range: 22–150 month). Patients were stratified into low-risk and high-risk groups based on risk factors (tumor size; lymph nodes and stromal invasion depth). Clinicopathologic characteristics of both groups are presented in Table 1. Within the low-risk group of 845 patients, 320 underwent LRH and 525 underwent OARH. There was three deaths and seven recurrences in the LRH cohort and four deaths and eight recurrences in the OARH cohort. Between the LRH and OARH cohorts, this groups showed no significant differences (all *P* > 0.05) in age, postoperative histologic subtype, LVSI, surgical margin, or adjuvant therapy.

**Table 1:** The clinicopathologic characteristics of patients in the two groups

	Low risk group			High risk group		
	LRH	OARH	*P*	LRH	OARH	*P*
	(*N* = 320)	(*N* = 525)		(*N* = 355)	(*N* = 926)	
Age (year)	47.87 ± 8.878	47.97 ± 9.647	*0.881*	48.53 ± 9.406	49.54 ± 9.543	*0.088*
Histologic type			*0.214*			*0.760*
Squamous cell	262 (81.9)	438 (83.4)		305 (85.9)	784 (84.7)	
Adenocarcinoma	51 (15.9)	83 (15.8)		40 (11.3)	118 (12.7)	
Adenosquamous	7 (2.2)	4 (0.8)		10 (2.8)	24 (2.6)	
Stromal invasion			*–*			*0.117*
Superficial	320 (100)	525 (100)		86 (24.2)	177 (19.1)	
Deep	–	–		217 (61.1)	595 (64.3)	
Unreported	–	–		52 (14.6)	154 (16.6)	
LVSI			*0.855*			*0.868*
Negative	297 (92.8)	489 (93.1)		266 (74.9)	698 (75.4)	
Positive	23 (7.2)	36 (6.9)		89 (25.1)	228 (24.6)	
Parametrium			*–*			*1.000*
Negative	320 (100)	525 (100)		352 (99.2)	916 (98.9)	
Positive	–	–		3 (0.8)	10 (1.1)	
Surgical margin			*0.470*			*0.842*
Negative	316 (98.8)	515(98.1)		350 (98.6)	916 (98.9)	
Positive	4 (1.3)	10(1.9)		5 (1.4)	10 (1.1)	
Pelvic lymph nodes						*0.000*
Negative	320 (100)	525 (100)	*–*	289 (81.4)	832 (89.8)	
Positive	–	–		66 (18.6)	94 (10.2)	
Adjuvant therapy			*0.278*			*0.275*
None	227 (70.9)	353 (67.2)		71 (20.0)	198 (21.4)	
Chemotherapy	82 (25.6)	159 (30.0)		155 (43.7)	359 (38.8)	
Radiotherapy/ radiochemotherapy	11 (3.4)	13 (2.5)		129 (36.3)	369 (39.8)	
FIGO stage (2018)			*-*			*0.468*
IA1–IB1	320 (100)	525 (100)		88 (24.8)	248 (26.8)	
≥ IB2	–	–		267 (75.2)	678 (73.2)	

Italics are used to represent the *P*-value, a common convention in statistics. This differentiation helps distinguish the p-value from other data points, highlighting its importance in statistical analysis

Values are presented as mean ± standard deviation or number (%)

*OARH* open abdominal radical hysterectomy, *LRH* laparoscopic radical hysterectomy, *LVSI* lymphovascular space invasion, *FIGO 2018* Federation International of Gynecology 2018

In high-risk group, there were 1281 patients, 355 underwent LRH and 926 underwent OARH. The LRH cohort experienced 29 deaths and 51 recurrences, while the OARH cohort reported 45 deaths and 89 recurrences. Significant differences were noted between the LRH and OARH cohorts in pelvic lymph node involvement (*P* < 0.000). However, the groups showed no significant differences (all *P* > 0.05) in age, postoperative histologic subtype, stromal invasion, LVSI, parametrial involvement, surgical margins, adjuvant therapy, or FIGO stage.

### Survival Analysis

**Fig. 2:**
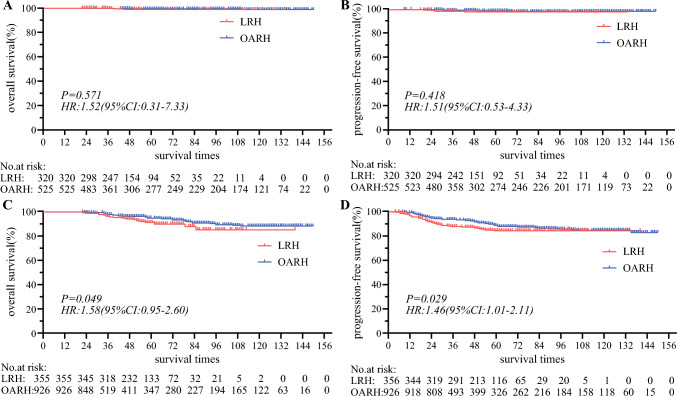
Survival outcomes between LRH and OARH groups: **A** Overall survival curves of the patients in low-risk group. **B** Progression-free survival curves of the patients in low-risk group. **C** Overall survival curves of the patients in high-risk group. **D** Progression-free survival curves of the patients in high-risk group

**Fig. 3:**
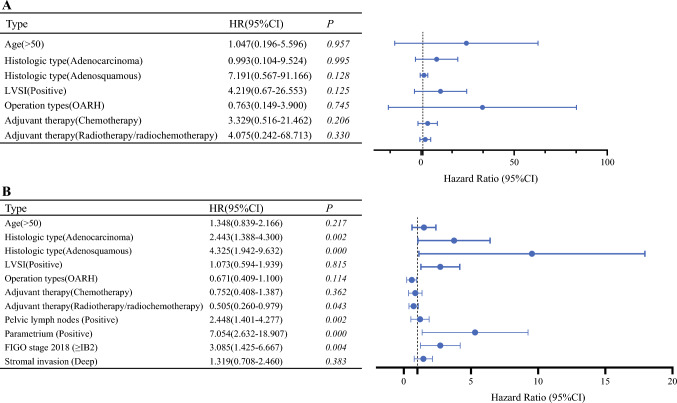
Multivariable Cox proportional hazards regression analysis with overall survival: **A** low risk group and **B** high risk group. Solid points represent point estimates and black lines represent 95% confidence intervals for each level of the interaction. Gray dotted line represents a hazard ratio of 1. *LVSI* lymphovascular space invasion, *FIGO 2018* Federation International of Gynecology 2018

**Fig. 4:**
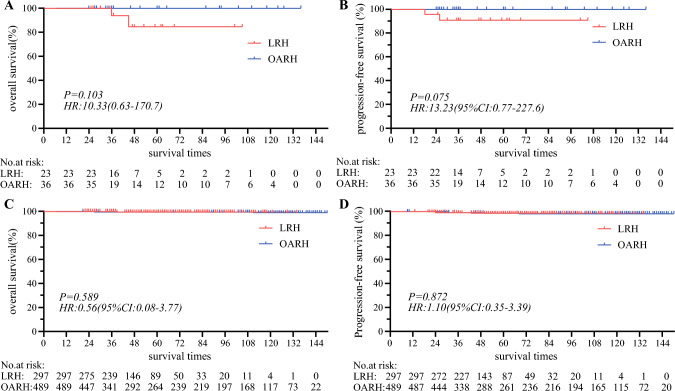
Survival outcomes between LRH and OARH in low-risk group based on the LVSI status. **A** Overall survival curves of the patients with positive LVSI. **B** Progression-free survival curves of the patients with positive LVSI. **C** Overall survival curves of the patients with negative LVSI. **D** Progression-free survival curves of the patients with negative LVSI

Among the low-risk group, LRH demonstrated comparable 5-year OS [98.6% versus 99.3%, *P* = 0.571, hazard ratio (HR): 1.52, 95% CI: 0.31–7.33, Fig. 2A] and 5-year PFS (97.6% versus 98.4%, *P* = 0.418, HR: 1.51, 95% CI: 0.53–4.33, Fig. 2B) to OARH. Multivariate Cox regression analysis showed that age, histologic subtype, LVSI, operation types, and adjuvant therapy were not significantly associated with overall survival (Fig. 3A). We then performed a stratified analysis based on the LVSI status. In the LVSI positive cohort, 23 individuals experienced LRH, with two deaths and two recurrences. Among the 36 patients who underwent OARH, there were 0 deaths and 0 recurrences. Additionally, LRH with positive LVSI showed 5-year OS (84.7% versus 100%, *P* = 0.103, HR: 10.33, 95% CI: 0.63–170.7, Fig. 4A) and PFS (90.9% versus 100%, *P* = 0.075, HR:13.23, 95% CI: 0.77–227.6, Fig. 4B) that were comparable with OARH. In the LVSI negative cohort, 297 individuals underwent LRH, with one death and five recurrences. Among the 489 patients who received OARH, there were 4 deaths and 8 recurrences. Furthermore, LRH with LVSI negative showed 5-year OS (99.5% versus 99.2%, *P* = 0.589, HR: 0.56, 95% CI: 0.08–3.77, Fig. 4C) and PFS (98.1% versus 98.3%, *P* = 0.872, HR: 1.10, 95% CI: 0.35–3.39, Fig. 4D) similar to those of OARH.

However, in the high-risk group, the 5-year overall survival for the LRH cohort was markedly lower at 91.3% compared with 94.8% for the OARH cohort (*P* = 0.049, HR: 1.58, 95% CI: 0.95–2.60, Fig. 2C). Similarly, the LRH cohort’s 5-year PFS was significantly lower at 84.0% compared with 88.8% for the OARH cohort (*P* = 0.029, HR: 1.46, 95% CI: 1.01–2.11; Fig. 2D). Following multivariate Cox regression analysis, histologic subtype, pelvic lymph nodes, parametrial involvement, adjuvant therapy, and FIGO stages were independent prognostic factors for overall survival in this group (Fig. 3B).

### Adverse Events

In the study population, 56 patients encountered postoperative complications: 25 in the low-risk group, 31 in the high-risk group. Forty-two patients experienced intraoperative transfusion, equally distributed between the two groups. For patients in the low-risk group, postoperative surgical complications occurred in 2.2% of the patients who underwent LRH as compared with 3.4% who underwent OARH (*P* = 0.298). Complications within the LRH cohort comprised: fistula (0.6%), ileus (0.3%), infection (0.6%), pelvic lymphocele with infection (0.3%), and urinary retention (0.3%). Conversely, the OARH cohort experienced complications: ileus (1.0%), infection (0.4%), poor wound healing (1.0%), deep venous thrombosis (0.2%), and pelvic lymphocele with infection (1.0%). The incidence of intraoperative transfusion was lower in the LRH than in the OARH (0.3% versus 3.8%; *P* = 0.002).

In the high-risk group, rates of postoperative surgical complications were comparable between the LRH and OARH groups (3.7% versus 1.9%, *P* = 0.073). The incidence of infection was higher in the LRH group than in the OARH group post-surgery (1.4% versus 0.2%; *P* = 0.02). Complications within the LRH cohort comprised: fistula (0.6%), ileus (0.6%), and pelvic lymphocele with infection (1.1%). Conversely, the OARH cohort experienced complications: fistula (0.1%), ileus (0.2%), poor wound healing (0.4%), and pelvic lymphocele with infection (1.0%). Intraoperative transfusion rates were 0.6% for patients undergoing LRH, compared to 2.1% for those undergoing OARH (*P* = 0.060) (Table 2).

**Table 2:** Adverse events details

	Low risk group			High risk group		
	LRH (N=320)	OARH (N=525)	*P*	LRH (N=355)	OARH (N=926)	*P*
Postoperative complications	7 (2.2)	18 (3.4)	*0.298*	13 (3.7)	18 (1.9)	*0.073*
Fistula	2 (0.6)	0 (0)	*0.143*	2 (0.6)	1 (0.1)	*0.187*
Ileus	1 (0.3)	5 (1.0)	*0.417*	2 (0.6)	2 (0.2)	*0.308*
Infection	2 (0.6)	2 (0.4)	*0.636*	5 (1.4)	2 (0.2)	*0.020*
Poor wound healing	0 (0)	5 (1.0)	*0.163*	0 (0)	4 (0.4)	*0.581*
Deep venous thrombosis	0 (0)	1 (0.2)	*1.000*	0 (0)	0 (0)	*–*
Pelvic lymphocele with infection	1 (0.3)	5 (1.0)	*0.417*	4 (1.1)	9 (1.0)	*0.762*
Urinary retention	1 (0.3)	0 (0)	*0.379*	0 (0)	0 (0)	*–*
Intraoperative transfusion						
Blood transfusion	1 (0.3)	20 (3.8)	*0.002*	2 (0.6)	19 (2.1)	*0.060*

Italics are used to represent the *P*-value, a common convention in statistics. This differentiation helps distinguish the p-value from other data points, highlighting its importance in statistical analysis

*OARH* open abdominal radical hysterectomy, *LRH* laparoscopic radical hysterectomy

## Discussion

Two recent multicenter retrospective analyses have evaluated the differences between LRH and OARH for early-stage, low-risk cervical cancer, characterized by tumors < 2 cm, no LVSI, an invasion depth of < 10 mm, and no lymph node involvement.^18,19^ These research findings corroborate our own, confirming the safety and efficacy of LRH in patients with low-risk cervical cancer. But, in contrast to the aforementioned studies, in “low-risk” cervical cancer, we emphasize that stromal invasion of less than half the cervical thickness rather than invasion depth of < 10 mm is more accurately defined as the superficial stromal invasion. Participants characterized by stromal invasion of the cervix greater than 10 mm but less than half of the cervical thickness, with no other high-risk elements, are designated as belonging to the low-risk category. Nonetheless, the low-risk group which includes such patients still demonstrates a favorable survival prognosis.

Other retrospective trials have raised concerns about the safety of laparoscopy. Juliana Rodriguez et al.^20^ and the SUCCOR study^21^ indicate that in early-stage cervical cancer patients, laparoscopy increases the risk of relapse and death compared with laparotomy. However, both included a certain proportion of high-risk populations (Juliana Rodriguez et al.: > 26.5%; SUCCOR study: > 56%), which might explain the increased risk of relapse and death associated with laparoscopy. Additionally, when stratified by tumor size, both studies showed that for patients with tumors smaller than 2 cm, laparoscopy did not present a significantly higher relapse and death risk compared with laparotomy. This is consistent with our findings.

However, to date, the only LACC trial^13^ that provides A level evidence, has shown a safety issue for laparoscopy. The authors explicitly state: “The results of this trial cannot be generalized to patients with ‘low-risk’ cervical cancer (tumor size of < 2 cm, no lymphovascular invasion, depth of invasion of < 10 mm, and no lymph-node involvement), because the trial was not powered to evaluate the oncologic outcomes of the two surgical approaches in that context” (Discussion, last sentence of the 7th paragraph). One reasons for this limitation is that the LACC trial included only 144 low-risk patients (superficial invasion: Open 61 pt; MIS 83 pts). In contrast, our study includes 845 patients in the low-risk group, all from a real-world setting, providing data that more accurately reflective actual clinical practice. Our study serves as a valuable supplement to the LACC trial. Nonetheless, we believe that further prospective studies are necessary to conclusively establish the safety of minimally invasive surgery (MIS) for patients with early-stage, low-risk cervical cancer.

The publication of the SHAPE Trial, a phase 3 international randomized trial, compared the 3-year pelvic recurrence rate and the risk of urinary complications between simple hysterectomy and radical hysterectomy for patients with early-stage low-risk cervical cancer.^22^^]^ The results of the SHAPE trial indicate that in this subset of radical hysterectomy patients, the 3-year pelvic recurrence rate was 2.17% with 5.5% urinary complications, while the group that underwent simple hysterectomy had a recurrence rate of 2.52% with 2.4% urinary complications. In this trial, over 70% of the radical hysterectomies performed were minimally invasive, suggesting that minimally invasive surgery for hysterectomy in the early-stage low-risk CC is relatively safe. Compared with the SHAPE Trial, our study demonstrated a 0.3% incidence of urinary complications and a total recurrence rate of 1.7% (15 out of 845 patients). Based on our research, Querleu–Morrow Type B radical hysterectomies can provide low urinary complications and recurrence rates, while also offering long-term survival. Therefore, we consider laparoscopic Querleu–Morrow Type B radical hysterectomies to be an ideal option for patients with early-stage, low-risk cervical cancer. From another perspective, the current SHAPE trial results are based on a 3-year follow-up period. Long-term outcomes are still under observation and require further follow-up.

In our country, there are approximately 100,000 new cervical cancer cases annually, accounting for about one-sixth of the world’s annual new cervical cancer cases.^1^ Our hospital is the largest cancer treatment center in the central-southern region, and cervical cancer is the most common type of gynecologic tumor. Surgeons need to undergo more than 10 years of MIS training. The high annual incidence of cervical cancer and the extensive training in MIS have increased the proficiency of surgeons in using MIS, thereby providing assurance for performing laparoscopic Querleu–Morrow Type B radical hysterectomy in early-stage, low-risk cervical cancer.

Moreover, the aforementioned retrospective studies^18,19^ and ConCerv Trial^23^ have integrated LVSI negative into the criteria for defining low-risk cervical cancer. However, in the SHAPE study, the group of patients with low-risk cervical cancer also included more than 12% patients who were LVSI positive. The inclusion of LVSI negative in the criteria defining low-risk cervical cancer remains clinically uncertain. When we performed a stratified analysis of the LVSI factor within the low-risk group. The results indicated that there were no significant differences in 5-year PFS and OS between LRH and OARH for both LVSI negative and positive patients. Therefore, we believe it is more suitable to exclude the LVSI negative from the definition of low-risk CC. This implies that for patients with early-stage, low-risk cervical cancer, the appropriate surgical approach can be chosen based on preoperative enhanced magnetic resonance imaging (MRI) and diffusion-weighted imaging (DWI) MRI, which is clinically feasible.

Preoperative evaluation of lymph node metastasis in cervical cancer primarily relies on commonly used imaging modalities: CT, enhanced MRI, DWI-MRI, or PET-CT. This multimodal approach ensures that lymph node assessments are as accurate as possible before surgical intervention. Among these modalities, MRI shows a sensitivity of 56% and a specificity of 91%.^24^ Recent studies, including a meta-analysis by Shen et al. suggest that DWI-MRI can improve the sensitivity and specificity of lymph node metastasis evaluation to 86% and 84%, respectively.^25^ Additionally, in 2017, the National Comprehensive Cancer Network (NCCN) upgraded the recommendation level of sentinel lymph node biopsy (SLNB) in cervical cancer treatment to 2A. For early-stage, low-risk cervical cancer patients, SLNB can be considered for lymph node evaluation.

For detecting stromal infiltration, conventional MRI is a reliably capable with a sensitivity of 87% and a specificity of 91%.^26^ Additionally, MRI offers the highest accuracy for assessing cervical tumor size, achieving a prediction precision of 93%.^27^ Enhanced MRI is the preferred imaging method for assessing local tumor extension in cervical cancer due to its excellent soft tissue resolution. It allows for the accurate evaluation of tumor size, infiltration, and pelvic lymph node enlargement in patients with cervical cancer. In summary, we can identify “low-risk” cervical cancer using enhanced MRI and DWI-MRI before surgery.

The strengths of our study: firstly, in low-risk early-stage cervical cancer, LRH demonstrates survival outcomes comparable to OARH. Conversely, in patients with high-risk, early-stage cervical cancer, LRH is associated with inferior 5-year OS and PFS rates, which correspond with those from the LACC study. Secondly, through large samples analysis, this study for the first time suggests defining superficial stromal invasion in “low-risk” cervical cancer as cervical penetration of less than half the depth, which is more accurate than invasion depth of < 10 mm. Thirdly, for patients with early-stage low-risk cervical cancer, we performed Querleu–Morrow type B radical hysterectomy by laparoscopy. These procedures led to just a 0.3% incidence of urinary complications, while the total recurrence rate was a mere 1.7% (15 out of 845). Therefore, for early-stage, low-risk patients with cervical cancer, the use of laparoscopic Querleu–Morrow type B radical hysterectomy not only ensures very low rate of urinary-related and other complication but also maintains a reduced rate of recurrence. Finally, we are able to identify “low-risk” cervical cancer by enhanced MRI and DWI-MRI pre-surgery and provide the best surgical approach—Querleu–Morrow type B radical hysterectomy by laparoscopy.

In the high-risk group, LRH is associated with poorer 5-year PFS and 5-year OS, which may be related to the surgical approach used. However, we discovered a statistically significant difference in lymph node involvement between the LRH cohort and the OARH cohort. Since lymph node involvement is one of the factors affecting prognosis, the poorer prognosis observed in the LRH within the high-risk group may be attributed to a higher proportion of patients with positive lymph nodes. In LACC trial, the majority of the data originates from high-risk patients (exceeding 70%), and these studies have substantiated that among patients with the high-risk factors, recurrence and mortality rates associated with LRH are higher compared to OARH. Thus, based on the findings of the LACC trial and the data from this study, we recommend open abdominal radical hysterectomy for patients in the high-risk group.

In conclusion, for patients in the low-risk group, we recommend laparoscopic Querleu–Morrow type B radical hysterectomy±pelvic lymphadenectomy. Furthermore, the appropriate surgical approach can be chosen based on preoperative enhanced MRI and DWI-MRI.
